# Atrial High‐Rate Episodes Mirror Atrial Fibrillation in Stroke Prediction: Evidence From an Indian Prospective Cohort

**DOI:** 10.1002/joa3.70370

**Published:** 2026-07-03

**Authors:** Sedhupathi Shanmugam, Sreevilasam P. Abhilash, Jyothi Vijay, Sapna erat Sreedharan, Narayanan Namboodiri

**Affiliations:** ^1^ Department of Cardiology Sree Chitra Tirunal Institute for Medical Science and Technology Thiruvananthapuram India; ^2^ Department of Neurology Sree Chitra Tirunal Institute for Medical Science and Technology Thiruvananthapuram India

**Keywords:** anticoagulation, atrial high‐rate episodes, CHA_2_DS_2_‐VASc, Indian population, stroke risk, subclinical AF

## Abstract

**Aims:**

To assess the risk of stroke/TIA and progression to atrial fibrillation (AF) in Indian patients with device‐detected atrial high‐rate episodes (AHRE) lasting less than 24 h.

**Methods and Results:**

In this single‐centre prospective matched cohort study, 109 patients with AHRE < 24 h matched to 109 controls without AHRE on age (+/−5 years), sex, device type, device indication, and CHA2DS2‐VASc score. Followed for a minimum of 12 months (mean 18.4 months). The CHA2DS2‐VASc score was near‐perfectly matched between groups (mean 3.26 vs. 3.29, score distribution chi‐square *p* = 1.00). A stroke or TIA occurred in 7 patients with AHRE versus 1 control (annual rate 4.28% vs. 0.61%; OR 7.41, 95% CI 0.90–61.3; *p* = 0.020; HR 7.19, 95% CI 0.89–58.48; log‐rank *p* = 0.031). Progression to clinical AF, AHRE ≥ 24 h, atrial flutter, or atrial tachycardia happened in 32 AHRE patients versus 2 controls (annual rate 19.7% vs. 1.2%; OR 22.2, 95% CI 5.17–95.6; HR 19.55, 95% CI 4.6–81.71; log rank *p* < 0.001). Stroke risk increased with CHA_2_DS_2_‐VASc score: 2.15% (score 3), 4.16% (score 4), 6.66% (score 5), and 9.5% (score ≥ 6).

**Conclusion:**

In this hypothesis‐generating study, device‐detected AF lasting less than 24 h is associated with a significantly higher risk of stroke/TIA and progression to clinical AF in Indian patients. It requires confirmation in larger studies. Region‐specific absolute risks must be considered when applying international anticoagulation guidelines.

## Graphical abstract

This prospective matched cohort study, which generates hypotheses, finds that device‐detected AF lasting under 24 h is linked to a notably increased risk of stroke/TIA and progression to clinical AF among Indian patients.

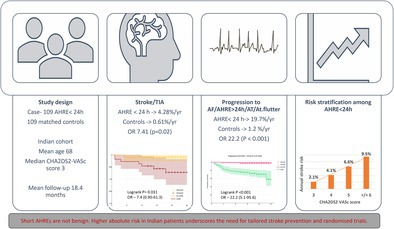

## 1 Introduction

Atrial fibrillation is the most common arrhythmia, often silent and diagnosed after a stroke. It's a main cause of cardioembolic stroke and can cause cryptogenic strokes, which account for 30%–40% of all strokes [1]. Hidden atrial fibrillation can now be detected early with cardiac implantable electronic devices (CIEDs) with atrial lead, called atrial high‐rate episodes (AHRE) or subclinical atrial fibrillation (SCAF), allowing for prevention through anticoagulation. These episodes are considered significant according to ESC 2020 guidelines when the rate exceeds 175 beats per minute and lasts more than 5 min [2]. Although AHRE lasting more than 24 h has been considered equivalent to clinical atrial fibrillation and is strongly linked to increased stroke risk, thereby warranting anticoagulation, the clinical significance of AHRE lasting less than 24 h remains uncertain [2]. In patients with AHRE < 24 h, stroke risk increases with higher CHA2DS2‐VAS scores, reaching an annual stroke rate of 3.78% when the score exceeds 2 [3]. AHRE < 24 h increases stroke risk by 2.5 times after adjusting for other risk factors [3]. However, the absolute stroke risk with AHRE < 24 h increases by only 1 percentage point per year, which is half the risk increase seen with clinical atrial fibrillation. Therefore, it is debatable whether to anti‐coagulate those with AHRE< 24 h, considering the bleeding risk associated with anticoagulation [4]. Hence, the anticoagulation in patients with AHRE < 24 h and a higher CHA2DS2‐VASc score is based on a clinical judgment considering risk and benefits; hence, our study started in May 2023 with the aim of identifying the stroke risk associated with AHRE < 24 h in the Indian population, which can help make the clinical judgment. In the meantime, two studies were published in 2023 with contradicting results. ARTESIA showed apixaban decreased the stroke risk in AHRE patients < 24 h compared to aspirin, but with increased bleeding risk [4]. NOAH‐AFNET was terminated prematurely by the Data and Safety Monitoring Board because edoxaban did not decrease the stroke risk compared to placebo, and instead increased the composite death and bleeding risk [5]. Currently, as per ESC 2024, it is a class IIB recommendation for anticoagulation for SCAF when the bleeding risk is low [6]. Against this backdrop of clinical uncertainty and limited South Asian data, the present study was designed as a hypothesis‐generating investigation into the risk of stroke and AF progression associated with AHRE < 24 h in an Indian population.

## 2 Materials and Methods

### 2.1 Study Population

This was a single‐centre, prospective, matched‐cohort study of patients with documented atrial high‐rate episodes, conducted from May 2023 to June 2025. Patients visiting our device clinic with atrial lead pacemakers, dual‐chamber implantable cardioverter‐defibrillators (ICDs), or cardiac resynchronisation therapy (CRT) devices, aged over 50 years, who were found to have atrial high‐rate episodes (AHREs) lasting less than 24 h on device interrogation, were enrolled as cases. Detection thresholds vary by manufacturer: ≥ 190 bpm (Medtronic), ≥ 180 bpm (Abbott), and ≥ 200 bpm (Biotronik/Boston Scientific), and usually last ≥ 5–6 min. Controls were matched on age (+/− 5 years), sex, device type, and indication for device implantation, with the primary objective of achieving a balanced CHA2DS2‐VASc score between groups—the internationally validated composite predictor of stroke risk. Since diabetes mellitus, hypertension, vascular disease, and sex each carry a weight of 1 point, while prior stroke/TIA and age > 75 years each carry 2 points, matching was performed to ensure that the overall composite score was equivalent, thereby directly balancing stroke risk between cases and controls by design. Individual component imbalances in single‐point variables, such as diabetes mellitus and hypertension, are expected and acceptable under composite score matching, as their net contribution to stroke risk is captured and balanced through the matched CHA2DS2‐VASc score total. Controls did not have documented AHRE. Patients excluded were those who had AHRE occurring within 2 months of device implantation, any history of atrial fibrillation (AF) or atrial flutter, or the presence of atrial tachycardia, or those who were already receiving or were initiated on oral anticoagulant (OAC) therapy at the time of enrollment. A total of 109 consecutive cases and controls were included from the pacemaker/device clinic at our institute between May 2023 and June 2024, with follow‐up continuing until June 2025. All patients completed at least 1 year of follow‐up.

### 2.2 Data Collection

At enrollment in the study, baseline data such as age, sex, height, weight, BSA, and BMI were collected. Details of cardiac comorbidities, including diabetes, hypertension, chronic kidney disease, coronary artery disease/ACS/AMI, peripheral artery disease, dyslipidemia, history of prior stroke/TIA, heart failure, CHA2DS2‐VASc score, smoking, alcohol use, obesity, OSA, COPD, inflammatory disease, thyroid disease (hypothyroidism), and valvular heart disease, were recorded. Details of medication use, including beta blockers, amiodarone, flecainide, propafenone, sotalol, digoxin, CCB, RAAS inhibitors, diuretics, statins, metformin, SGLT2 inhibitors, and antiplatelets, were noted. Information regarding device interrogation, including the type of cardiac implantable device (CEID), the indication for the device, and details of atrial high‐rate episodes, was documented.

### 2.3 Outcomes

Patients were followed for at least 1 year. Primary outcomes included (1) ischemic stroke/TIA (Transient ischemic attack), (2) atrial high‐rate episodes that progress to atrial fibrillation or its equivalents, such as episodes lasting longer than 24 h, atrial flutter, or atrial tachycardia. Secondary outcomes comprised (1) acute coronary syndrome or myocardial infarction, (2) hospitalization for heart failure, and (3) death.

### 2.4 Statistical Analysis

After data collection, the data were compiled using Microsoft Excel and analysed with SPSS software version 30. All variables were tested for normality with the Shapiro–Wilk test. Normally distributed continuous variables were reported as mean and standard deviation, while non‐normally distributed variables were shown as median and interquartile range. All categorical variables are presented as frequencies and proportions. Significance tests included the unpaired *t*‐test, chi‐square test, Fisher's exact test, and Mann–Whitney U test. For event‐free analysis, the Kaplan–Meier curve with the log‐rank test was used. Univariate and multivariate hazard ratios were calculated using Cox regression analysis. Results were considered statistically significant if the *p* was < 0.05 within 95% confidence intervals. To address residual confounding, a multivariable logistic regression adjusting for AHRE group, antiplatelet use, diabetes mellitus, and CHA2DS2‐VASc score was performed as a supplementary analysis. A landmark sensitivity analysis and device type subgroup analysis were also conducted; these are reported in the Supporting Information S1, S3–S5.

## 3 Results

**Baseline characteristics:** A total of 109 cases and controls were enrolled, with baseline characteristics presented in Table 1, showing a well‐matched age and gender distribution. The mean age was 67.91 years (SD 9.29) for cases and 67.57 (SD 8.51) for controls. Both groups had 65.14% males. Follow‐up duration was similar, averaging 18.41 months (SD 3.95), median 18, IQR 15–22. BMI averaged 25.36 (SD 3.8) in cases and 25.25 (SD 3.3) in controls. Crucially, the CHA2DS2‐VASc score‐ the primary matching variable and the internationally validated composite predictor of stroke risk‐ was near perfectly matched between groups: mean 3.26 (SD 1.53) in cases and 3.29 (SD 1.56) in controls, with both groups having a median of 3, IQR 2–4, and a score distribution that was virtually identical across all strata (chi‐square *p* = 1.000). This matching directly balanced the composite stroke risk between groups by design. Since prior stroke/TIA and age > 75 years each carry a weight of 2 points in the CHA2DS2‐VASc system, while diabetes mellitus, hypertension, vascular disease, and sex each carry 1 point, matching was weighted to prioritize equivalence of these higher‐weight components as well as the composite total; observed imbalances in individual single‐point components are therefore expected and do not represent residual confounding for the stroke outcome. LAVI was higher in cases (mean 29.46, SD 5.47; median 28, IQR 25–34) compared to controls (mean 26.83, SD 4.4; median 26, IQR 23–30), and this difference was statistically significant. This elevation is a pathophysiologically expected finding, as AHREs represent a form of atrial tachyarrhythmia that causes progressive atrial remodeling and consequent atrial enlargement, rather than an independent source of confounding. The maximum values were 46 and 41; the minimum values were 19 and 20. LVEF median was 60 (IQR 55–64) in cases and 62 (IQR 59–64) in controls.

**TABLE 1:** Baseline demographic and clinical characteristics of AHRE cases compared to controls.

Baseline variables	Case (109)	Control (109)	*p*
Age (mean +/− std. dev)	67.91 (+/− 9.29)	67.57 (+/−8.51)	0.779
Male (%)	71 (65.14)	71 (65.14)	1
Total follow‐up month (median & IQR)	18 (15–22)	18 (15–22)	1
BMI (median & IQR)	25 (23–27.2)	25.2 (23.12–26.9)	0.817
CHA2DS2‐VASc score (median & IQR)	3 (2–4)	3 (2–4)	0.867
LAVI (median & IQR)	29 (25–34)	26 (23–30)	< 0.001*
LVEF (median & IQR)	60 (55–64)	62 (59–64)	0.122

*Note:* Std dev, Standard deviation; IQR, Interquartile range; BMI, Body Mass Index; (calculated using weight in kilograms divided by height in meters squared); CHA2DS2 VASc score, a score used to estimate the annual stroke risk in patients with non‐valvular atrial fibrillation; ranging from 0 to 9. The higher the score, the greater the risk of stroke. The score includes congestive heart failure/left ventricular dysfunction, hypertension (> 140/90), age 65 to 75, diabetes mellitus, vascular disease (myocardial infarction, aortic plaque, peripheral artery disease), and sex category (female), which are assigned a score of 1. Age > 75 and prior stroke or transient ischemic attack (TIA) are assigned a score of 2. LAVI, left atrial volume index; calculated by measuring left atrial volume using the modified Simpson's biplane method from 2D transthoracic echocardiography, then indexed to body surface area (square root of [height in centimeters × weight in kilograms] divided by 3600). LVEF, left ventricular ejection fraction; calculated from 2D transthoracic echocardiography using the Simpson's biplane method.

**Device types and indications** are listed in Table 2, showing a perfect match between the case and control groups with no statistical difference (*p* = 1). The most common device was the dual‐chamber pacemaker (67.8%), followed by atrial single‐chamber pacemakers (15.6%), CRT‐P and CRT‐D (each 7.3%), and the dual‐chamber implantable cardioverter (1.8%). The primary indications were sinoatrial node dysfunction (56.8%) and atrioventricular block (26.6%). Other indications included prophylaxis for ventricular tachycardia, ventricular dys‐synchrony, and severe LV dysfunction.

**TABLE 2:** Device type and implantation indications in AHRE cases versus controls.

Device and indications	Case *n* (%) 109	Control *n* (%) 109	Total *n* (%) 218	*p*
Device				
DC PPI	74 (67.89)	74 (67.89)	148 (67.89)	1
AAI PPI	17 (15.6)	17 (15.6)	34 (15.6)	
DC ICD	2 (1.83)	2 (1.83)	4 (1.83)	
CRT P	8 (7.34)	8 (7.34)	16 (7.34)	
CRT D	8 (7.34)	8 (7.34)	16 (7.34)	
Indication				
SA node disease‐Sick sinus syndrome	62 (56.88)	62 (56.88)	124 (56.88)	1
AV node disease‐ Heart block	29 (26.61)	29 (26.61)	58 (26.61)	
VT primary prophylaxis	1 (0.92)	1 (0.92)	2 (0.92)	
VT secondary prophylaxis	2 (1.83)	2 (1.83)	4 (1.83)	
Ventricular Dys synchrony	9 (8.26)	9 (8.26)	18 (8.26)	
Severe LV dysfunction	6 (5.5)	6 (5.5)	12 (5.5)	

Abbreviations: AAI PPI, atrial permanent pacemaker implantation; AV node disease, atrioventricular node disease; CRT‐D, cardiac resynchronisation therapy‐defibrillator; CRT‐P, cardiac resynchronisation therapy‐pacemaker; DC ICD, dual chamber implantable cardioverter defibrillator; DC PPI, dual chamber permanent pacemaker implantation; LV, left ventricle; SA node disease, sinoatrial node disease; VT, ventricular tachycardia.

**A comparison of comorbidities** between the case and control groups is presented in Table 3. As described in the baseline characteristics section above, the CHA2DS2‐VASc score was adequately and near‐perfectly matched between groups (mean 3.26 vs. 3.29, *p* = 0.867; score distribution chi‐square *p* = 1.000), directly controlling composite stroke risk by design. The high‐weight components (prior stroke/TIA and age > 75 years, each 2 points) were well matched: prior stroke/TIA in 14 (12.8%) cases vs. 12 (11.01%) controls (*p* = 0.676), and age > 75 years in 25 (22.9%) cases vs. 20 (18.3%) controls (*p* = 0.386). Observed imbalances in individual single‐point components, such as diabetes mellitus and hypertension, are expected and do not reflect residual confounding, as their contributions to stroke are absorbed within the matched composite score. The distribution of individual comorbidities is as follows:

**TABLE 3:** Distribution of comorbidities in AHRE cases versus controls*.

Comorbdities	Case *n* (%) 109	Control *n* (%) 109	Total *n* (%) 218	*p*
Prior stroke/TIA	14 (12.84)	12 (11.01)	26 (11.93)	0.676
HTN	91 (83.49)	86 (78.9)	177 (81.19)	0.386
DM	46 (42.20)	60 (55.05)	106 (48.17)	0.078
CKD	14 (12.84)	10 (9.17)	24 (11.01)	0.387
CAD/ACS/CCS	32 (29.36)	32 (29.36)	64 (29.36)	1
POVD	1 (0.92)	1 (0.92)	2 (0.92)	1
DLP	38 (34.86)	34 (31.19)	72 (33.03)	0.565
History of HF	24 (22.02)	26 (23.85)	50 (22.94)	0.747
Smoking	22 (20.18)	19 (17.43)	41 (18.81)	0.603
Alcohol	25 (22.94)	21 (19.27)	46 (21.1)	0.507
OSA	12 (11.01)	5 (4.59)	17 (7.8)	0.077
Obesity	51 (46.79)	54 (49.54)	105 (48.17)	0.684
COPD	11 (10.09)	6 (5.5)	17 (7.8)	0.207
Hypothyroidism	24 (22.02)	12 (11.01)	34 (15.6)	0.044*
Bio prosthetic valve	3 (2.75)	1 (0.92)	4 (1.83)	0.622

Abbreviations: ACS, Acute coronary syndrome; CAD, Coronary artery disease; CCS, Chronic coronary syndrome; CKD, Chronic kidney disease; COPD, Chronic obstructive pulmonary disease; DLP, Dyslipidemia; DM, Diabetes mellitus; History of HF, History of heart failure; HTN, Hypertension; OSA, Obstructive sleep apnea; POVD, Peripheral occlusive vascular disease; TIA, Transient ischemic attack.

* Those already diagnosed with the specific comorbidities by the treating physician are mentioned. Not screened for these conditions during the study.

Diabetes mellitus was present in 46 patients (42%) in the case group, fewer than the 60 patients (55%) in the control group, with a *p* of 0.078. Most of our study population in the case group was hypertensive, with 91 patients (83%), compared to 86 patients (79%) in the control group, with a *p* of 0.386, showing no significant difference between the groups. Chronic kidney disease (CKD) was present in 14 patients (13%) in the case group and 10 patients (9%) in the control group. Coronary artery disease (CAD), including acute and chronic coronary syndrome, was observed in 32 patients (29%) in both groups. Peripheral occlusive vascular disease (POVD) was seen in 1 patient (0.9%) in each group. Dyslipidemia affected 38 patients (35%) in the case group and 34 (31%) in the control. Prior heart failure was noted in 24 patients (22%) in the case group (including 19 with HFrEF and 5 with HFpEF) and 26 patients (23.8%) in controls (including 23 with HFrEF and 3 with HFpEF). Smoking was reported in 22 (20%) case patients and 19 (17.4%) controls. Alcoholism was present in 25 (23%) case and 21 (19%) control patients. Obstructive sleep apnea (OSA) was more common in cases (12 patients, 11.7%) than in controls (5 patients, 4.5%), with a *p* = 0.07. Obesity (BMI > 25) was present in 51 (46.7%) cases and 54 (49.5%) controls. COPD was more frequent among cases (11, 10%) than among controls (6, 5.5%), but the difference was not statistically significant (*p* = 0.207). Hypothyroidism was higher in cases (24, 22%) than in controls (12, 11%), and the difference was statistically significant (*p* = 0.044). Bioprosthetic valve replacement was seen in 3 cases and 1 control.

**A comparison of baseline medications** between the case and control groups is shown in Table 4. Among the cases, 32 patients (29.3%) were on antiplatelet therapy, fewer than 50 (45.8%) in the controls, with a *p* = 0.018. Eighty‐five (77.9%) cases used beta blockers, which was higher than in controls (42, 38.5%), *p* < 0.001. Twenty‐four (22%) were on thyroxine, higher than 12 (11%) in controls, *p* = 0.044. Thiazide diuretics were used by 10 cases (9.1%) and 12 controls (11%), *p* = 0.65. Loop diuretics were used by 29 (26.6%) cases and 28 (25.6%) controls, *p* = 0.878. 75 (68.81%) were on statins in both groups, *p* = 1.0. MRA was used in 26 (23.8%) cases and 20 (18.8%) controls; *p* = 0.319. 61 (55.9%) in cases and 62 (56.8%) in controls were on calcium channel blockers, *p* = 0.891. 56 (51.3%) were on ACEi, ARB, or neprilysin inhibitors, fewer than 67 patients (61.4%) in controls, *p* = 0.133. 24 (22%) were on SGLT2 inhibitors, compared to 22 (19.2%) in controls, *p* = 0.616. 24 (22.0%) cases and 5 (4.9%) controls were on amiodarone, *p* < 0.001. 5 (4.5%) cases and 4 (3.6%) controls were on digoxin; *p* = 0.65.

**TABLE 4:** Comparison of baseline medication use between AHRE cases and controls.

Medications	Case *n* (%) 109	Control *n* (%) 109	Total *n* (%) 218	*p*
SAPT/DAPT	32 (29.36)	50 (45.87)	82 (37.61)	0.018*
Betablockers	85 (77.98)	42 (38.53)	127 (58.26)	0.286
Thiazide diuretics (CTD/HCT)	10 (9.17)	12 (11.01)	22 (10.09)	0.653
Loop diuretics	29 (26.61)	28 (25.69)	57 (26.15)	0.878
Statins	75 (68.81)	75 (68.81)	150 (68.81)	1
MRA	26 (23.85)	20 (18.35)	46 (21.1)	0.319
Thyroxine	24 (22.02)	12 (11.01)	36 (16.51)	0.044*
CCB	61 (55.96)	62 (56.88)	123 (56.42)	0.891
ACEI/ARB/ARNI	56 (51.38)	67 (61.47)	123 (56.42)	0.133
SGLT2i	24 (22.02)	21 (19.27)	45 (20.64)	0.616
Amiodarone	24 (22.0)	5 (4.9)	29 (13.3)	< 0.001*
Digoxin	5 (4.5)	4 (3.6)	9 (4.1)	0.65

Abbreviations: ACEi, angiotensin‐converting enzyme inhibitor; ARB, angiotensin receptor blocker; ARNI, angiotensin receptor‐neprilysin inhibitor; CCB, calcium channel blocker; CTD, chlorthalidone; DAPT, dual antiplatelet; HCT, hyrochlorothiazide; MRA, mineralocorticoid receptor antagonist; SAPT, single antiplatelet; SGLT2i, sodium‐glucose co‐transporter 2 inhibitor.

**FIGURE 1:**
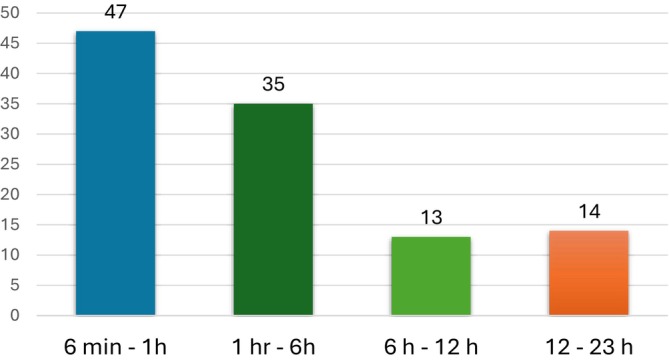
Baseline AHRE burden. Abbreviations: Min; minutes; h, hours; AHRE, Atrial high‐rate episodes.

**AHRE burden at inclusion**: Figure 1 shows that among cases with documented atrial high‐rate episodes (AHRE), they were grouped into four categories based on the longest AHRE burden at study inclusion, graded from 1 to 4: 6 min to 1 h (grade 1), 1 to 6 h (grade 2), 6 to 12 h (grade 3), and 12 to 24 h (grade 4). The figure indicates most (47) were in group 1, followed by 35 in group 2, 13 in group 3, and 14 in group 4.

At a mean follow‐up of 18.4 months, among the 47 patients in grade 1 at enrollment, 19.1% (9) progressed to grade 2, 6.4% (3) to grade 3, 12.8% (6) to grade 5, and 61.7% (29) remained in grade 1. Of the 35 patients at grade 2 at enrollment, 17.1% (6) progressed to grade 3, 34.2% (12) to grade 5, and 48.5% (17) remained at grade 2. Among the 13 patients in grade 3 at enrollment, 53.8% (7) progressed to grade 5, while 46.1% (6) remained in grade 3. Among the 14 patients in grade 4 at enrollment, 50% (7) progressed to grade 5, and 50% (7) remained in grade 4. Overall, 54.6% (59) of patients remained at their initial grade at a mean follow‐up of 18.4 months, while 45.3% (49) progressed to a higher AHRE burden (Table 5).

**TABLE 5:** Incidence of transition to higher AHRE burden at a mean follow‐up of 18.41 months (among the case group *n* = 109).

	Incidence of transition to higher AHRE burden at a mean follow‐up of 18.41 months			
Progression	6 min–< 1 h	1–< 6 h	6–< 12 h	12–< 23 h
Transition to ≥ 1 h	19.1%			
Transition to ≥ 6 h	6.4%	17.1%		
Transition to ≥ 12 h				
Transition ≥ 24 h/AF	12.8%	34.2%	53.8%	50.0%

Abbreviations: AF, atrial fibrillation; h, hour; min, minutes; n, number.

**FIGURE 2:**
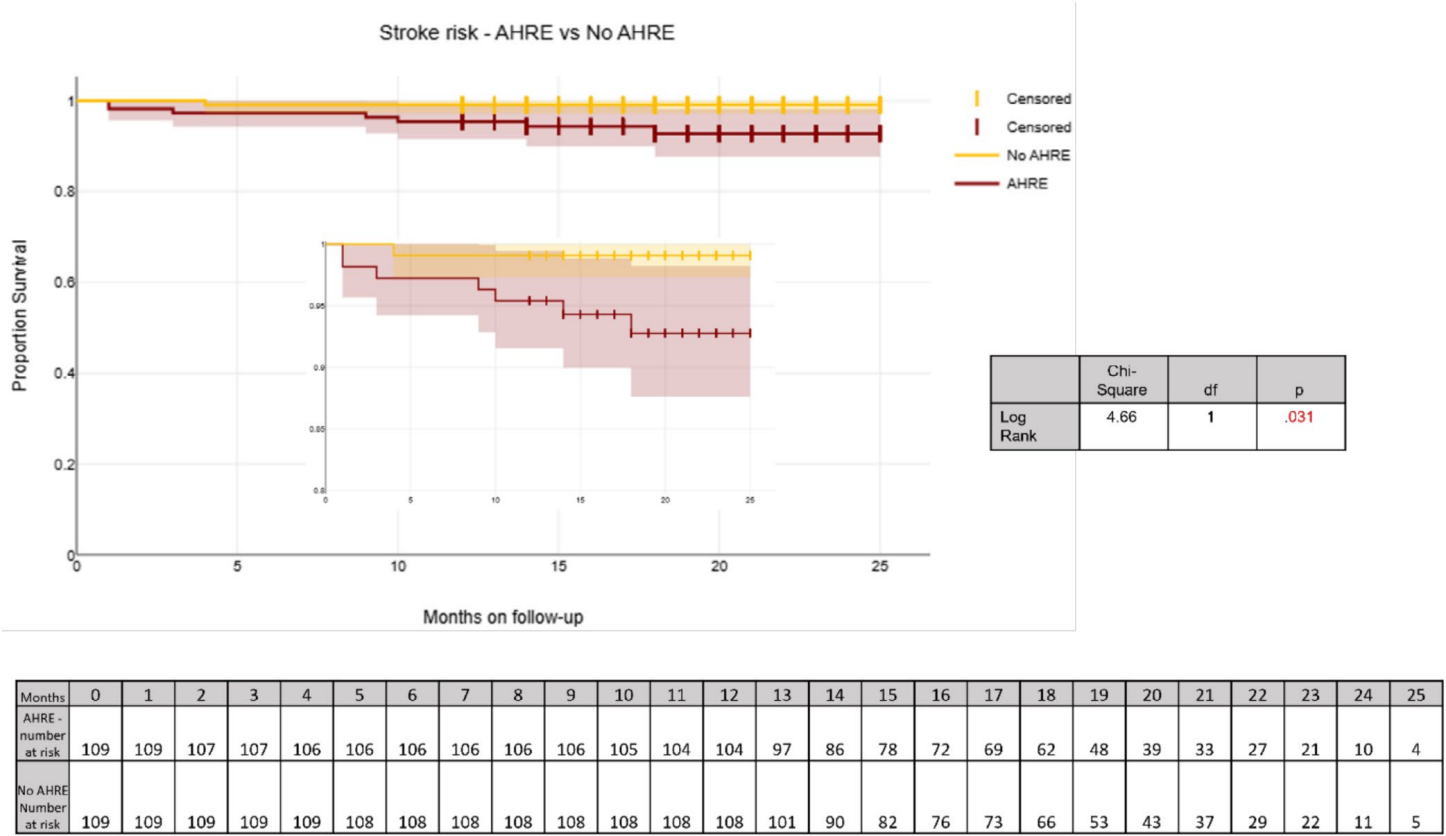
Kaplan–Meier survival analysis comparing stroke‐free survival between the AHRE < 24 h and the No AHRE groups. Event‐free analysis for stroke risk, comparing patients with AHRE less than 24 h and controls, using the Kaplan–Meier curve, revealed a statistically significant difference. Log‐rank analysis yielded a *p* of 0.031, indicating statistical significance.

**FIGURE 3:**
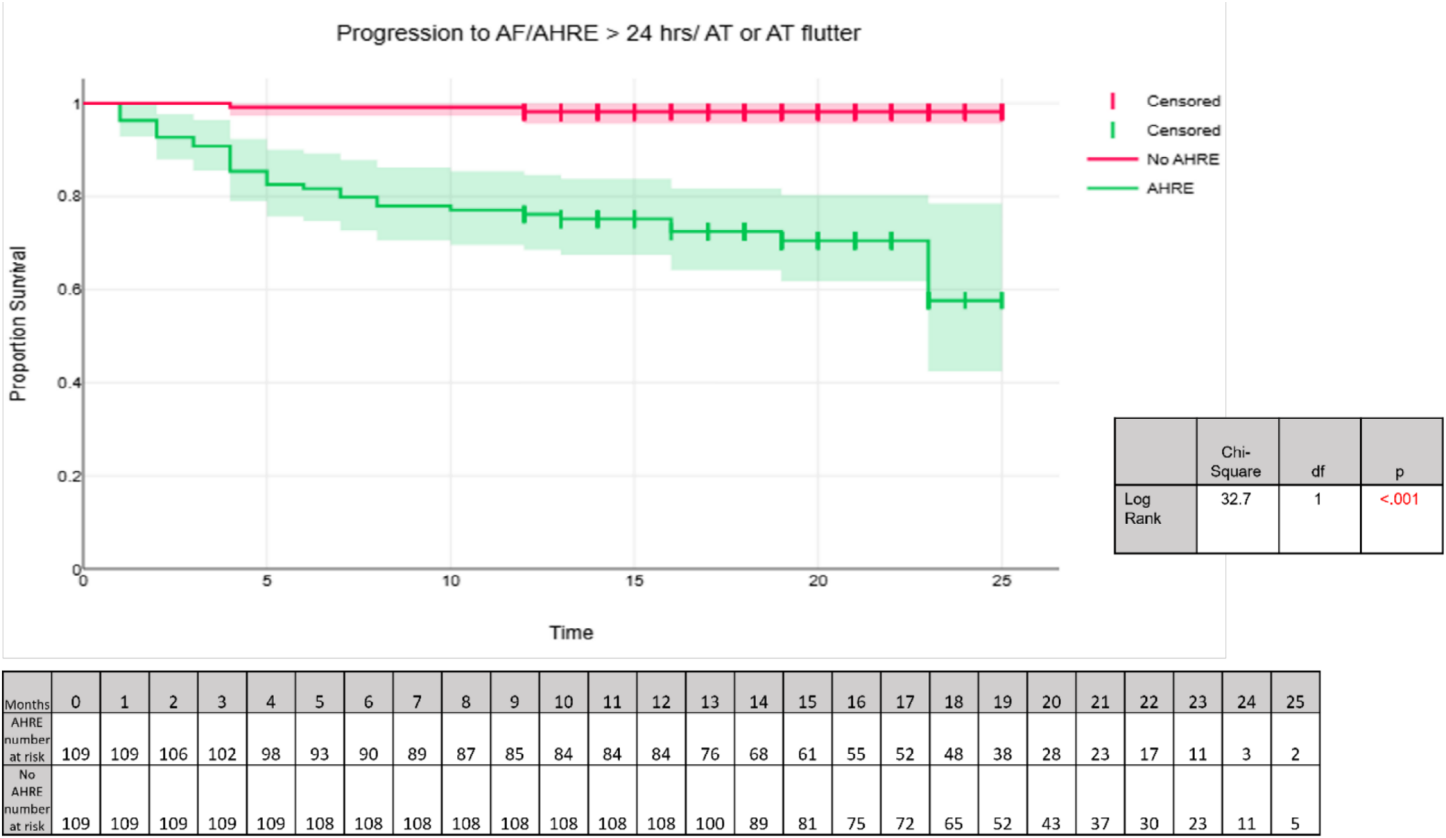
Kaplan–Meier survival analysis showing progression to AF/AHRE > 24 h/AT or atrial flutter in AHRE vs. No AHRE groups. Event‐free analysis using the Kaplan–Meier curve for progression to clinical AF and its equivalents comparing patients with AHRE < 24 h and controls revealed a statistically significant difference; log‐rank analysis yielded a *p* < 0.01, indicating statistical significance.

**Primary outcomes:** (1) Among the case and control groups, who were followed for a mean duration of 18.4 months, ischemic stroke or Transient Ischemic Attack (TIA) was observed in 7 patients (5 strokes and 2 TIA) in the case group (patients with AHRE—atrial high rate episodes) compared to 1 patient in the control group (patients without AHRE). The odds ratio was 7.41 (95% CI, 0.90–61.3; logistic regression, *p* = 0.020) (Table 6). Cox proportional hazards regression yielded a hazard ratio (HR) of 7.19 (95% CI 0.89–58.48; *p* = 0.065). The Cox *p* reflects the limited event count (*n* = 8), while the log‐rank test, the pre‐specified test for Kaplan–Meier analysis, confirmed a statistically significant difference (*p* = 0.031; Figure 2). Multivariable‐adjusted analysis (Table S2) yielded an adjusted OR of 5.31 (95% CI, 0.58–48.81). All three effect estimates are directionally consistent, indicating an approximately 5–7fold excess stroke/TIA risk in AHRE patients. The annual stroke/TIA rate among patients with AHRE < 24 h is 4.28% per year. (2) Incidence of AF/AHRE > 24 h/AT or atrial flutter during a mean follow‐up of 18.4 months, Case vs. Control—Among 109 patients in the case group (patients with AHRE lasting less than 24 h) 32 patients (29.3%) progressed to clinical atrial fibrillation (AF) or its equivalents, such as AHRE lasting more than 24 h, atrial tachycardia (AT), or atrial flutter. (Twenty‐one developed AF, six developed AHRE over 24 h, and five developed AT or atrial flutter.) The event rate was 19.7% per year, significantly higher than in the control group (patients without AHRE), in which only 2 patients developed clinical atrial fibrillation, yielding an event rate of 1.22% per year. The odds ratio was 22.2 (95% CI 5.17–95.58, *p* < 0.001). Cox proportional hazards regression yielded a hazard ratio of 19.55 (95% CI, 4.68–81.71; *p* < 0.0001), confirming a highly significant and robust association (Table 6). Event‐free analysis using the Kaplan–Meier curve revealed a statistically significant difference, and the log‐rank test yielded a *p* < 0.01 (Figure 3). At 18 months, 72.5% of AHRE cases remained free of AF progression compared to 98.1% of controls (Table 6). (Figure 3).

**TABLE 6:** Incidence of new cardiovascular events and outcomes in patients with AHRE < 24 h versus controls.

Events (Outcomes)	Case	% per year	Control	% per year	Odds ratio (95% conf. interval)	*p*	Hazard ratio (95% CI)	*p*
AF	21 (19.27)	12.8	2 (1.83)	1.22	22.2 (5.17–95.58)		19.55 (4.68–81.7)	< 0.001*
AHRE > 24 h	6 (5.5)	3.6	0 (0)	—		< 0.001*		
AT/Atrial flutter	5 (4.59)	3.3	0 (0)	—				
Stroke	5 (4.59)	3.06	1 (0.92)	0.61	5.19 (0.6–45.2)	0.212	5.10 (0.60–43.61)	0.137
Stroke/TIA	7 (6.42)	4.28	1 (0.92)	0.61	7.4 (0.90–61.3)	0.02 * (Log rank 0.031)	7.19 (0.89–58.48)	0.065
MI/ACS	6 (5.5)	3.6	4 (3.67)	2.44	1.52 (0.42–5.58)	0.748	1.53 (0.43–5.42)	0.511
HF Hospitalization	6 (5.5)	3.6	6 (5.5)	3.6	1.0 (0.31–3.2)	1.00	1.01 (0.33–3.14)	0.982
Death	2 (1.83)	1.22	1 (0.92)	0.61	2.03 (0.18–22.39)	1.00	1.99 (0.18–21.99)	0.573

Abbreviations: ACS, acute coronary syndrome; AF, atrial fibrillation; AHRE > 24 h, atrial high‐rate episodes over 24 h; AT, atrial tachycardia; HF, heart failure; MI, myocardial infarction; TIA, transient ischemic attack.

**Secondary outcomes (**Table 6 **):** Over a mean follow‐up of 18.4 months, 6 patients in the case group developed ACS (3.6% per year) compared to 4 in the control group (2.44% per year); OR 1.52 (*p* = 0.517, not significant). Both groups experienced 6 hospitalisations for heart failure (3.6% per year, OR 1, *p* = 1). In the case group, 2 patients died (one from sudden cardiac arrest, one from heart failure; 1.22% per year), while 1 death (from sudden cardiac arrest; 0.61% per year) occurred in controls; OR 2.03 (*p* = 1, not significant).

**CHA2DS2‐VASc Score and Stroke Distribution in Patients with AHRE < 24 h (Case group):** The CHA2DS2‐VASc score is used to predict stroke risk in patients with non‐valvular atrial fibrillation, with scores ranging from 0 to 9. In our study of 109 patients in the case group (patients with AHRE < 24 h), the minimum CHA2DS2‐VASc score was 0, seen in 2 patients, and the maximum was 7, also seen in 2 patients. The mean score was 3.26, with an interquartile range (IQR) of 2 to 4. Twelve patients had a score of 1, 21 patients had a score of 2, the majority had a score of 3 (31 patients), 16 patients had a score of 4, 20 patients had a score of 5, and 5 patients had a score of 6 (Figure 4A). Over a mean follow‐up of 18.4 months, one patient with a score of 2 (out of 21) experienced a transient ischemic attack (TIA). One patient with a score of 3 (out of 31) experienced a stroke. One patient with a score of 4 (out of 16) experienced a stroke. Among 20 patients with a score of 5, two developed strokes, and one had a TIA. One patient with a score of 6 (out of 5) experienced a stroke (Figure 4B). Therefore, from our study, the annual stroke risk for AHRE < 24 h related to scores 3, 4, 5, and ≥ 6 is 2.15%, 4.16%, 6.66%, and 9.5%, respectively (Table 7).

**FIGURE 4A:**
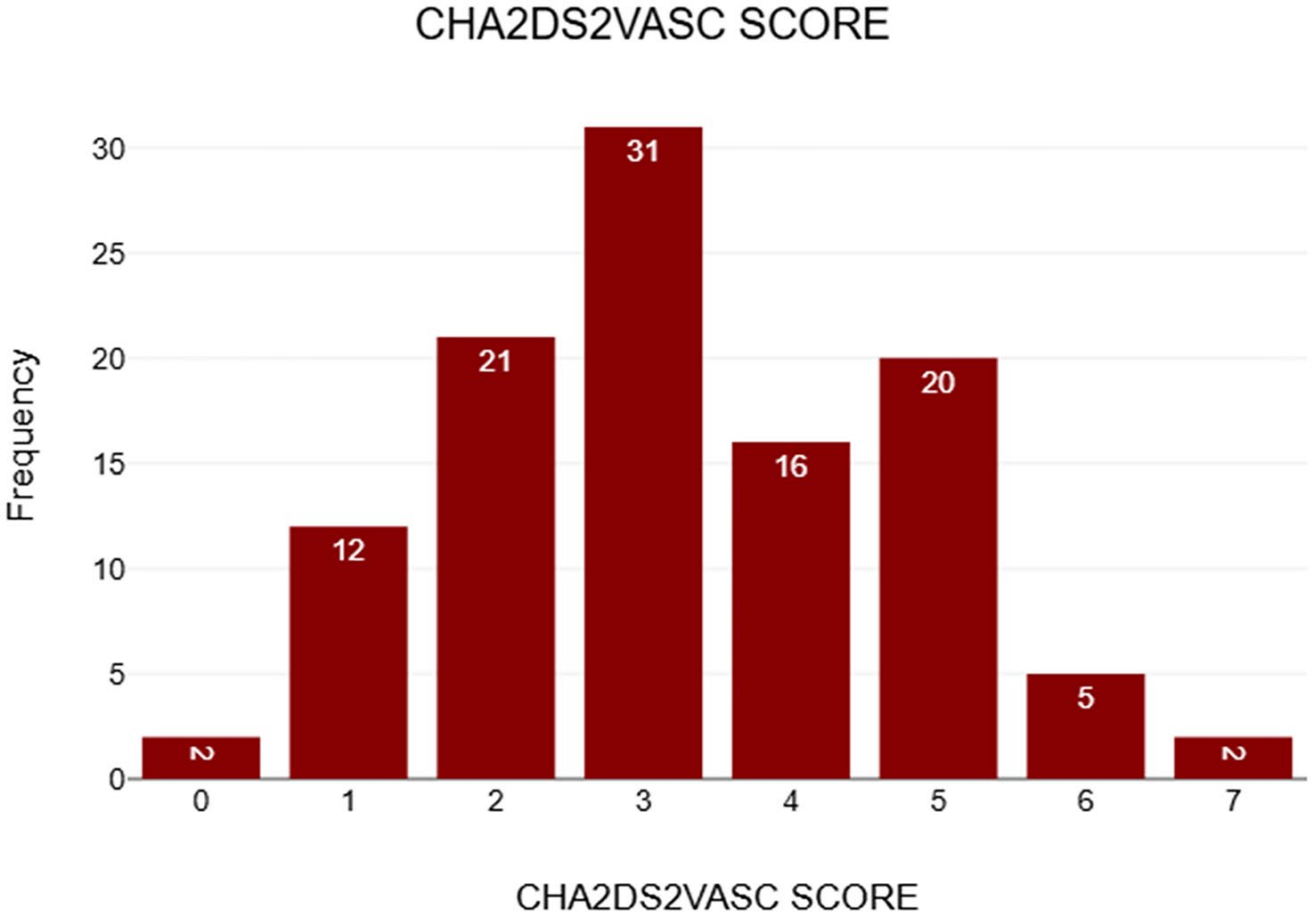
Distribution of CHA_2_DS_2_‐VASc scores among the case group (AHRE < 24 h). 4A‐ Among 109 patients in the case group (AHRE < 24 h), most (31) had a CHA2DS2‐VASc score of 3, followed by 21 with a score of 2, 20 with a score of 5, 16 with a score of 4, 12 with a score of 1, 5 with a score of 6, 2 with a score of 0, and 7.

**FIGURE 4B:**
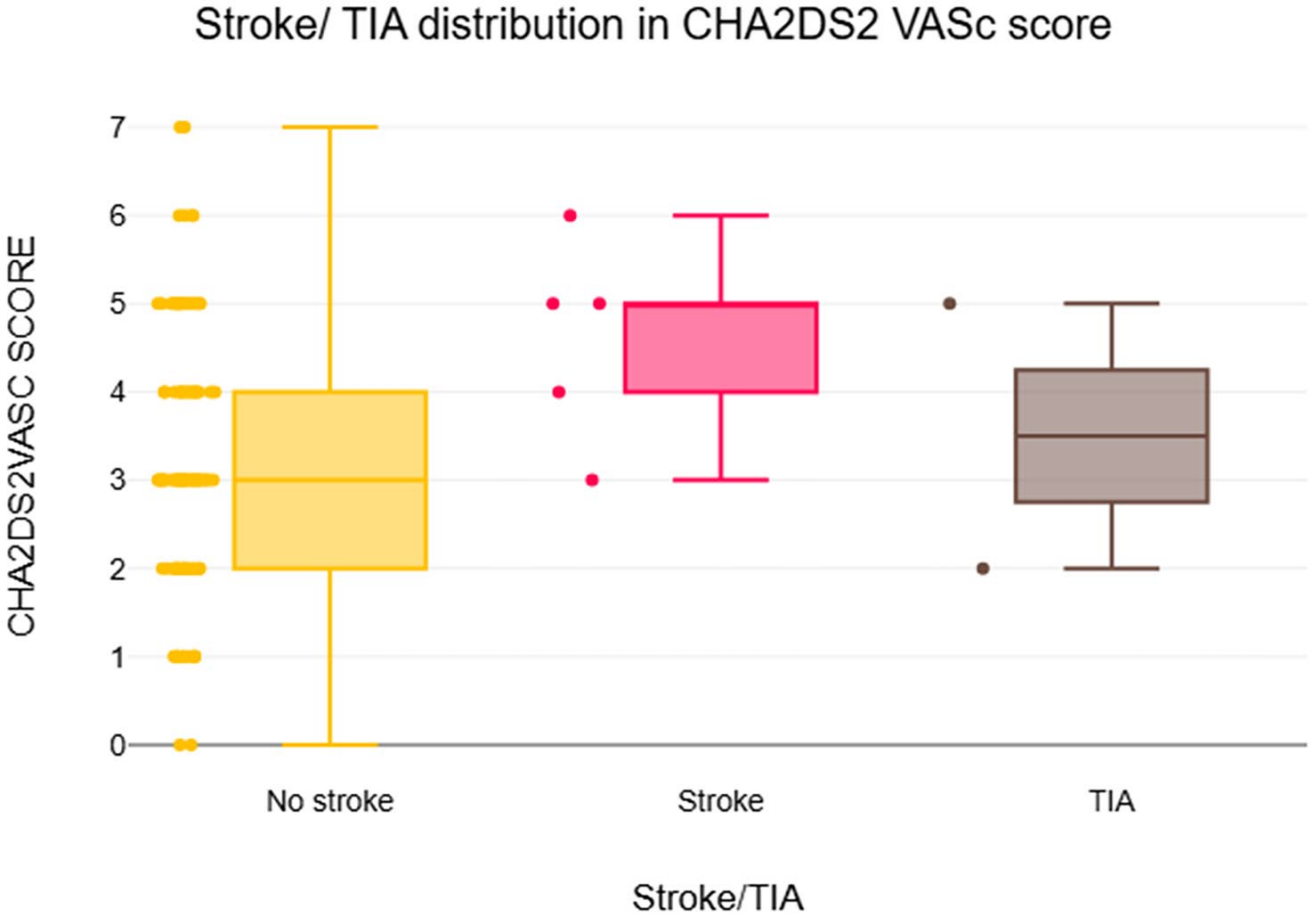
CHA_2_DS_2_‐VASc score stratified by stroke/TIA outcomes among the case group (AHRE < 24 h). 4B‐ One patient had strokes in each CHA2DS2‐VASc score category of 3, 4, and 6. Two with scores of 5 had strokes, and one with a score of 2 had a TIA. Another with a score of 5 had a TIA.

**TABLE 7:** Annual stroke and stroke/TIA rates by CHA_2_DS_2_‐VASc category in patients with AHRE < 24 h.

Annual stroke/TIA rate as per CHA2DS2‐VASc score category—among AHRE < 24 h from our study		
CHA2DS2‐VASc score	Stroke risk	Stroke/TIA risk
0	—	—
1	—	—
2	—	3.1%
3	2.15%	—
4	4.16%	—
5	6.66%	10%
≥ 6	9.5%	—

Abbreviation: TIA, Transient Ischemic Attack.

## 4 Discussion

In this prospective matched cohort study among Indian patients with cardiac implantable devices, we found that those with atrial high‐rate episodes (AHRE) < 24 h had a significantly higher risk of stroke or TIA (annual incidence approximately 4.28%) compared to matched control patients (~0.61%), with an odds ratio of about 7.41 (95% CI 0.90 to 61.3; logistic regression, *p* = 0.020). These results should be interpreted with caution given the wide confidence intervals, which reflect the limited stroke/TIA event count (*n* = 8): these results are hypothesis‐generating rather than definitive. Additionally, roughly one‐third of the AHRE group progressed to overt clinical AF or sustained atrial arrhythmias during an 18‐month follow‐up. These findings highlight that even “short” device‐detected atrial tachyarrhythmias are not benign in a high‐risk population.

In comparison with prior studies, the ASSERT trial was pivotal in demonstrating that subclinical atrial tachyarrhythmias (episodes lasting more than 6 min) detected by pacemakers or defibrillators were associated with approximately a 2.5‐fold increase in stroke or systemic embolism over approximately 2.5 years of follow‐up in a primarily Western population [3]. In ASSERT, the observed annual rate of stroke/systemic embolism in patients with device‐detected subclinical AF was approximately 1.69%, compared to about 0.89% in those without; therefore, our observed rate is higher, likely due to differences in baseline risk factors (such as CHA_2_DS_2_‐VASc scores and comorbidities) and potentially ethnic or population‐specific factors.

Several meta‐analyses have reinforced the increased stroke risk associated with subclinical AF (SCAF)/AHRE. Mahajan et al. reported an annual stroke incidence of approximately 1.89 per 100 person‐years, with about a 2.4‐fold increased risk compared to those without SCAF [7]. Noseworthy et al. (in a 2019 pooled analysis) showed a 2.4‐fold increase in stroke risk associated with subclinical AF (95% CI 1.8–3.3) [8]. Meng et al. (2023) [9] found that cumulative AHRE burden correlated more strongly with stroke risk than the duration of the longest episode and demonstrated that a high AHRE burden was associated with a 2.52‐fold increased risk of stroke [9]. Vitolo et al., in their meta‐analysis, showed that device‐detected AHRE was associated with a relative risk of 2.13 for thromboembolic events and 3.34 for incident clinical AF [10]. These findings collectively highlight the consistency of increased risk across various settings.

Our study shows a higher absolute risk in an Indian cohort, possibly due to elevated rates of diabetes, hypertension, and coronary artery disease among our patients. This supports the idea that comorbidity burden increases stroke risk in device‐detected AHRE.

The recent randomized trials ARTESIA and NOAH‐AFNET, conducted among patients with device‐detected atrial high‐rate episodes and a CHA2DS2‐VASc score of 2 or higher, provided conflicting evidence regarding the benefit of anticoagulation. ARTESIA showed that apixaban reduced ischemic stroke but increased bleeding risk compared to aspirin [4], whereas NOAH‐AFNET compared edoxaban and placebo, which was stopped early due to lack of efficacy and increased adverse events [5].

A meta‐analysis of data from ARTESIA and NOAH‐AFNET shows that anticoagulation reduces the risk of ischemic stroke by 32%, but it increases the risk of major bleeding by 62%, although the risk of fatal bleeding remains low [11].

Accordingly, the 2024 ESC guidelines recommend that anticoagulation may be considered in patients with device‐detected subclinical AF when bleeding risk is acceptable (Class IIb, Level B) [6]. The 2017 EHRA consensus [12] already emphasized the variability in definitions of AHRE and the uncertainty of management in < 24 h episodes. Our findings support both that AHRE < 24 h carry a clinically meaningful risk, especially in high CHA_2_DS_2_‐VASc patients, and that decisions should remain individualized.

We compared the CHA_2_DS_2_‐VASc‐stratified annual stroke rates observed among our AHRE (< 24 h) patients with those reported in the large Swedish Atrial Fibrillation cohort. In the Swedish cohort, the annual ischemic stroke rates by CHA_2_DS_2_‐VASc were approximately: 0 → 0.2%, 1 → 0.6%, 2 → 2.2%, 3 → 3.2%, 4 → 4.8%, 5 → 7.2%, and 6 → 9.7% (with further increases for higher scores) [13].

In our AHRE cohort, the observed annual stroke rates by CHA_2_DS_2_‐VASc score were as follows: score 3, 2.15%; score 4, 4.16%; score 5, 6.66%; and score ≥ 6, 9.5% (Table 7). Thus, for lower to moderate scores (3–5), the stroke risk associated with AHRE in our Indian cohort is slightly lower but broadly comparable to the Swedish registry AF risk; for CHA_2_DS_2_‐VASc ≥ 6, the AHRE‐associated stroke risk in our cohort approaches the registry risk for clinical AF (9.5% vs. approximately 9.7% per year). These data imply that the stroke risk conferred by brief device‐detected AHRE is not negligible and—importantly—approximates the risk of clinical AF in patients with high CHA_2_DS_2_‐VASc scores. These estimates should be interpreted with caution, given the small numbers of events within each score stratum.

The clinical implications of this comparison are twofold. First, AHRE < 24 h in patients with higher CHA_2_DS_2_‐VASc scores (especially ≥ 5–6) presents an absolute stroke risk comparable to that seen in established AF cohorts and may warrant serious consideration of anticoagulation after an individualized bleeding‐risk assessment. Second, in intermediate CHA_2_DS_2_‐VASc strata (3, 4), the AHRE risk is close to, but slightly below, the estimates from the Swedish AF registry—supporting a nuanced, dynamic approach (such as enhanced surveillance and shared decision‐making rather than automatic anticoagulation). Combined with randomized trial data (ARTESIA, NOAH‐AFNET) and guideline recommendations, our findings suggest that arrhythmia burden plus CHA_2_DS_2_‐VASc should guide management decisions, and that region‐specific absolute risks must be taken into account when applying international guidelines.

Progression from AHRE to clinically manifest AF has important clinical implications, as it increases both thromboembolic risk and symptomatic burden. In our cohort, nearly one‐third of patients with AHRE < 24 h progressed to AF, AHRE ≥ 24 h, atrial flutter, or atrial tachycardia during a mean follow‐up of 18.4 months, corresponding to an annualized event rate of approximately 19.7%. This finding aligns with earlier reports. In the ASSERT trial, patients with AHRE had a more than fivefold higher risk of developing clinical AF compared to those without AHRE [3]. Similarly, the TRENDS study showed that patients with device‐detected atrial tachyarrhythmias had an increased likelihood of subsequent AF diagnosis and stroke risk [14]. Noseworthy et al. conducted a pooled analysis, showing a 5.7 to 5.9 times increased risk of progression to clinical AF [8]. A recent meta‐analysis by Mahajan et al. further reinforced the notion that AHRE presence strongly predicts incident AF, with relative risks ranging from 2.5 to 5.0, depending on the episode duration and burden [7]. ARTESIA and NOAH‐AFNET studies have demonstrated that a significant proportion of patients progressed to clinical atrial fibrillation, with 24.1% and 18.2% [6].

Compared to these studies, our data indicate a higher absolute progression rate to clinical AF, with an odds ratio of 22.2 and an event rate of 19.7% per year. The relatively short follow‐up duration in our study emphasises that progression from AHRE to clinical AF may occur early, highlighting the need for intensified rhythm monitoring and possibly earlier preventive measures in high‐risk patients.

Several methodological considerations merit discussion. First, although the composite stroke/TIA endpoint was significant (OR 7.41, 95% CI 0.90–61.3, *p* = 0.02 by logistic regression and Kaplan–Meier log‐rank *p* = 0.031). The Cox proportional hazards model yielded a consistent hazard ratio of 7.19 (95% CI 0.89–58.48; *p* = 0.065); the Cox *p* reflects the limited event count rather than a true absence of effect, as the log rank test‐ the pre‐specified test for Kaplan–Meier analysis‐ confirms a statistically significant difference. Stroke alone (5 cases vs. 1 control; OR 5.19, *p* = 0.097) did not reach significance due to the limited number of events (*n* = 6). TIA is part of the thromboembolic endpoint in landmark AHRE trials like ASSERT and ARTESIA, making its inclusion consistent with the literature. Second, cases and controls were matched based on CHA2DS2‐VASc scores‐ a validated stroke predictor‐ rather than individual comorbidities. The matching prioritized the composite score and high‐weight components, which were closely matched (prior stroke/TIA: 14 vs. 12, *p* = 0.676; age > 75: 25 vs. 20, *p* = 0.386). The mean scores were nearly identical (3.26 vs. 3.29, *p* = 0.867). Imbalances in components like diabetes and hypertension are expected and do not indicate residual confounding, as these are captured within the score. Third, LAVI was higher in cases (29.46 vs. 26.83 mL/m2, *p* < 0.001), reflecting atrial remodeling due to AHRE‐related atrial tachyarrhythmia, which causes atrial enlargement. Fourth, more controls received antiplatelet therapy (45.8% vs. 29.3%, *p* = 0.018), likely suppressing their stroke/TIA rate and potentially overestimating the OR. The adjusted OR 5.31 (*p* = 0.058), accounting for this is more conservative. Despite this, the annual stroke/TIA rate in cases (4.28%) was much higher than in ASSERT (1.69%), where many AHRE patients also used aspirin. Two stroke/TIA events occurred despite antiplatelet use in cases, showing it does not fully protect against thromboembolic events associated with AHRE. This aligns with ARTESIA, where OAC outperforms antiplatelets, and ASSERT, where antiplatelet use doesn't eliminate excess stroke risk. While the OR may be conservative, the elevated absolute stroke risk in AHRE patients remains significant and clinically relevant.

## 5 Limitations

Our study has limitations affecting interpretation. It is a single‐centre, observational study with a moderate sample size and 18‐month follow‐up; the primary outcome of stroke/TIA occurred in only 7 cases versus 1 control, resulting in a wide confidence interval (OR 7.41, 95% CI 0.90–61.3), limiting estimate precision. Findings are hypothesis‐generating rather than definitive. Although the CHA2DS2‐VASc score was matched between groups (mean 3.26 vs. 3.29, median 3 vs. 3, *p* = 0.867; score distribution *p* = 1.000), residual confounding may exist since no formal propensity score matching or IPTW was performed (Table S2). AHRE status was treated as a fixed exposure; control patients (2) developing de novo AHREs during follow‐up were analyzed separately, and excluding these did not alter primary results, though a time‐dependent covariate model was not feasible. Silent cerebral infarcts may be underdiagnosed without systematic neuroimaging. Higher antiplatelet use in controls likely suppressed their stroke/TIA rate, possibly overestimating the OR. The adjusted OR of 5.31 (95% CI, 0.58–48.81; Table S2), accounting for antiplatelet use, is more conservative. Larger multicentre, long‐term studies with standardized AHRE detection are needed.

## 6 Conclusion

In this hypothesis‐generating prospective matched‐cohort study, AHRE < 24 h was associated with a substantially higher risk of stroke/TIA (OR 7.41, HR 7.19, adjusted OR 5.31) and progression to clinical AF (OR 22.2, HR 19.55) among Indian patients. Confirmation in larger multicentre studies is required. Region‐specific absolute risks must be considered when applying international guidelines to South Asian AHRE patients.

**Input for Future Trial Design:** The high event rate in our cohort suggests Indian and South Asian patients are valuable for future anticoagulation trials.

## Author Contributions

S.S. (concept/design: Equal, data analysis/interpretation: Equal, drafting article: Equal, critical revision of article: Equal, approval of article: Equal, statistics: Equal, data collection: Equal); S.A. (concept/design: Equal, data analysis/interpretation: Equal, drafting article: Equal, critical revision of article: Equal, approval of article: Equal, statistics: Equal); J.V. (concept/design: Equal, data analysis/interpretation: Equal, drafting article: Equal, critical revision of article: Equal, approval of article: Equal, statistics: Equal); S.S. (concept/design: Equal, data analysis/interpretation: Equal, drafting article: Equal, critical revision of article: Equal, approval of article: Equal, statistics: Equal); N.N. (concept/design: Equal, data analysis/interpretation: Equal, drafting article: Equal, critical revision of article: Equal, approval of article: Equal, statistics: Equal, data collection: Equal).

## Funding

The authors have nothing to report.

## Disclosure

Changes From Original Submission: All revisions made in response to reviewer comments and data verification are marked using track changes throughout this manuscript. Deleted text is shown as strikethrough, and new or inserted text is shown as underlined text. A full list of all changes with reasons is provided in the response to Reviewers (Section III) and in Table S6.

## Ethics Statement

Clearance obtained from the Institute Ethical Committee.

## Consent

Obtained prior to enrollment in the study for participation and publication of results.

## Conflicts of Interest

The authors declare no conflicts of interest.

## Supporting information


**Table S1:** Consolidated list of Abbreviations.
**Table S2:** Multivariable Logistic Regression: Adjusted predictors of Stroke/TIA.
**Table S3:** Stroke/TIA outcomes stratified by device type.
**Table S4:** Landmark Sensitivity Analysis (Immortal Time Bias).
**Table S5:** Stroke alone Vs Stroke/TIA composite endpoint.
**Table S6:** CHA2DS2‐VASc score distribution: Matching Quality Verification.

## Data Availability

The data that support the findings of this study are available from the corresponding author upon reasonable request.
