# Supplementary material for: Atrial High‐Rate Episodes Mirror Atrial Fibrillation in Stroke Prediction: Evidence From an Indian Prospective Cohort
Source: J Arrhythm. 2026 Jul 3;42(4):e70370. doi: 10.1002/joa3.70370 (PMC13332316; doi:10.1002/joa3.70370)
Supplement: Supplementary file 1 — Table S1: Consolidated list of Abbreviations. Table S2: Multivariable Logistic Regression: Adjusted predictors of Stroke/TIA. Table S3: Stroke/TIA outcomes stratified by device type. Table S4: Landmark Sensitivity Analysis (Immortal Time Bias). Table S5: Stroke alone Vs Stroke/TIA composite endpoint. Table S6: CHA2DS2‐VASc score distribution: Matching Quality Verification. [file JOA3-42-e70370-s001.docx]

**SUPPLEMENTARY MATERIAL**

**Atrial High-Rate Episodes Mirror Atrial Fibrillation in Stroke Prediction: Evidence from an Indian Prospective Cohort**

**Sedhupathi Shanmugam, Sreevilasam P. Abhilash, Jyothi Vijay, Sapna Erat Sreedharan, Narayanan Namboodiri**

**Table S1- Consolidated list of Abbreviations**

All abbreviations used in the main manuscript, tables, and Figures are defined below and at first appearance in the main text, each table/figure legend

| Abbreviation | Full form |
| --- | --- |
| AHRE | Atrial high-rate episode |
| AF | Atrial Fibrillation |
| TIA | Transient Ischemic Attack |
| SCAF | Subclinical Atrial Fibrillation |
| CIED | Cardiac Implantable Electronic Devices |
| OAC | Oral Anticoagulant |
| SAPT | Single Antiplatelet therapy |
| DAPT | Dual Antiplatelet Therapy |
| CHA2DS2-VASc | Congestive heart failure, Hypertension, Age>/= 75 (x2), Diabetes, Stroke/TIA (x2), vascular disease, Age 65-74, Sex (Female) |
| LAVI | Left Atrial Volume Index (ml/m2) |
| LVEF | Left Ventricular ejection Fraction (%) |
| DM | Diabetes Mellitus |
| HTN | Hypertension |
| CKD | Chronic Kidney Disease |
| CAD | Coronary artery disease |
| ACS | Acute Coronary Syndrome |
| HF | Heart failure |
| HFrEF | Heart failure with Reduced Ejection fraction |
| HFpEF | Heart Failure with Preserved Ejection Fraction |
| OSA | Obstructive Sleep Apnea |
| COPD | Chronic Obstructive Pulmonary Disease |
| POVD | Peripheral Occlusive Vascular Disease |
| DLP | Dyslipidaemia |
| DC PPI | Dual Chamber Permanent Pacemaker Implantation |
| AAI PPI | Atrial Single Chamber Pacemaker Implantation |
| DC ICD | Dual Chamber Implantable Cardioverter Defibrillator |
| CRT-P | Cardiac Resynchronisation Therapy- pacemaker |
| CRT- D | Cardiac Resynchronisation Therapy- Defibrillator |
| OR | Odds Ratio |
| HR | Hazard Ratio |
| CI | Confidence interval |
| SD | Standard Deviation |
| IQR | Interquartile Range |
| MRA | Mineralocorticoid Receptor agonist |
| CCB | Calcium Channel Blocker |
| ACEi | Angiotensin- converting Enzyme inhibitor |
| ARB | Angiotensin Receptor Blocker |
| ARNI | Angiotensin receptor- Neprilysin Inhibitor |
| SGLT2i | Sodium- Glucose Co-transporter 2 Inhibitor |
| BMI | Body Mass Index |
| BSA | Body Surface Area |
| ESC | European Society of Cardiology |
| IPTW | Inverse Probability or Treatment Weighting |
| PSM | Propensity Score Matching |
| ASSERT | Asymptomatic Atrial fibrillation and Stroke evaluation in pacemaker Patients trial |
| ARTESIA | Apixaban for the Reduction of Thrombo-Embolism in Patients with Device-detected Sub-clinical Atrial Fibrillation |
| NOAH-AFNET | Non-Vitamin K Antagonist Oral Anticoagulants in Patients with Atrial High- Rate Episodes |

************************************************************************

**Table S2- Multivariable Logistic Regression: Adjusted predictors of Stroke/TIA**

Rationale: To address the potential confounding effect of antiplatelet imbalance, a multivariate logistic regression was performed with stroke/TIA as the outcome. Covariates: AHRE group, antiplatelet use (SAPT/DAPT), and CHA2DS2-VASc score. Note: (1) DM is not included as a separate covariate because its stroke risk contribution is fully captured within the CHA2DS2-VASc score, which was directly matched between groups. (2) LAVI is not included because its elevation in cases reflects the mechanistic consequence of AHRE- driven atrial remodelling, not an independent confounder. All 218 patients included, 8 total events.

| **Variable** | **Adj. OR** | **95% CI** | **p** | **Interpretation** |
| --- | --- | --- | --- | --- |
| **AHRE group (vs. no AHRE)** | **5.31** | 0.58 – 48.81 | **0.140** | Direction consistent with the unadjusted OR of 7.41. Attenuation expected with only 8 total events. Confirms the association is not fully explained by measured confounders. |
| Antiplatelet use (SAPT/DAPT) | 0.34 | 0.06 – 2.15 | 0.253 | The protective trend, with higher antiplatelet use in controls (45.8% vs 29.3%), may lead to a decreased stroke/TIA risk in them. |
| CHA₂DS₂-VASc score | 1.71 | 0.95 – 3.08 | 0.075 | Borderline significance; consistent with the score-based stroke gradient in the primary analysis. The score was directly matched between groups — included here for completeness. |

**Key Finding:** The adjusted OR for the AHRE group is 5.31 (95% CI 0.58–48.81)—a more conservative and reliable estimate that accounts for antiplatelet therapy imbalance. The unadjusted OR (7.41) might overstate the risk slightly because controls had higher antiplatelet use (45.8% compared to 29.3%), which lowers their stroke rate. Nonetheless, the absolute annual stroke/TIA rate of 4.28% in cases is still clinically significant: 2 out of 7 events occurred in patients already on antiplatelet therapy, and this rate is notably higher than the 1.69% observed in the ASSERT trial, despite similar antiplatelet coverage. Both odds ratios reveal a meaningful increased risk, but the adjusted OR should be used to interpret the main effect size.

************************************************************************

**Table S3- Stroke /TIA outcomes stratified by device type**

Rationale: AHRE detection thresholds vary across manufacturers. Device type was used as a proxy for manufacturer. Device type was perfectly matched between cases and controls (p=1.0, Table 2).

| **Device** | **Cases n** | **Events (Cases)** | **Controls n** | **Events (Controls)** | **OR (95% CI), p** |
| --- | --- | --- | --- | --- | --- |
| DC PPI | 74 | 5 (6.8%) | 74 | 0 (0%) | — |
| AAI PPI | 17 | 1 (5.9%) | 17 | 1 (5.9%) | 1.00 |
| DC ICD | 2 | 0 | 2 | 0 | — |
| CRT-P | 8 | 0 | 8 | 0 | — |
| CRT-D | 8 | 1 (12.5%) | 8 | 0 | — |
| **Total** | **109** | **7 (6.4%)** | **109** | **1 (0.9%)** | **7.41 (0.90–61.3), p=0.02** |

**Interaction p-value:** Device type × AHRE group: p = 0.837. No significant interaction — findings are consistent across device types and are not driven by manufacturer-specific detection thresholds.

************************************************************************

**Table S4 – Landmark Sensitivity Analysis (Immortal Time Bias)**

Rationale: Two control patients developed de novo AHRE that progressed to clinical AF during follow-up. Excluding them approximates a time-fixed exposure comparison.

| **Analysis** | **Case n** | **Events** | **Ctrl n** | **Events** | **OR** | **95% CI** | **p** |
| --- | --- | --- | --- | --- | --- | --- | --- |
| **Primary analysis (full cohort, n=218)** | 109 | **7** | 109 | **1** | **7.41** | 0.90–61.3 | **0.031** |
| Landmark (2 controls with de novo AF excluded) | 109 | **7** | 107 | **1** | **7.27** | 0.88–60.2 | **0.065** |

**Finding:** OR changes slightly (7.41 to 7.27) after exclusion, supporting the robustness of the primary finding against immortal time bias.

************************************************************************

**Table S5- Stroke alone Vs Stroke/TIA composite endpoint**

Rationale: Stroke alone was not significant, while the composite was. Both endpoints are presented explicitly for transparency.

| **Endpoint** | **Cases** | **Controls** | **OR** | **95% CI** | **p** | **Comment** |
| --- | --- | --- | --- | --- | --- | --- |
| **Stroke alone** | 5 (4.6%) | 1 (0.9%) | **5.19** | 0.60–45.2 | 0.097 | Not significant; 6 total events — severely underpowered. |
| **Stroke/TIA (composite)** | 7 (6.4%) | 1 (0.9%) | **7.41** | 0.90–61.3 | **0.020*** | Significant. TIA is an accepted endpoint in ASSERT, ARTESIA, and NOAH-AFNET. |

**Note:** *p-value from logistic regression (log rank p=0.031). TIA is an established thromboembolic endpoint in ASSERT, ARTESIA and NOAH-AFNET.

************************************************************************

**Table S6- CHA2DS2-VASc score distribution: Matching Quality Verification**

Cases and controls were matched to achieve equivalent CHA2DS2-VASc scores. The following table demonstrates the near-perfect score distribution matching achieved.

| **CHA₂DS₂-VASc Score** | **Cases (n=109)** | **Controls (n=109)** | **Difference/p-value** |
| --- | --- | --- | --- |
| Score 0 | 2 | 2 | 0 |
| Score 1 | 12 | 12 | 0 |
| Score 2 | 21 | 20 | 1 |
| Score 3 | 31 | 30 | 1 |
| Score 4 | 16 | 18 | 2 |
| Score 5 | 20 | 20 | 0 |
| Score 6 | 5 | 4 | 1 |
| Score 7 | 2 | 3 | 1 |
| **Total** | **109** | **109** | 6 |
| **Mean ± SD** | **3.26 ± 1.53** | **3.29 ± 1.55** | **p = 0.867** |
| **Median (IQR)** | **3 (2–4)** | **3 (2–4)** | **—** |
| **Score distribution (chi-square)** | **—** | **—** | **p = 1.000** |

**Statistical Confirmation:** Overall CHA2DS2-VASc score: Mann-Whitney p=0.867. Score distribution chi-square p=1.000. Total absolute difference across all score strata: Only 6 patients. This represents near-perfect 1:1 matching by composite stroke risk.

************************************************************************
